# Supplementary material for: Suicidal thoughts and behaviour among healthcare workers in England during the COVID-19 pandemic: A longitudinal study
Source: PLoS One. 2023 Jun 21;18(6):e0286207. doi: 10.1371/journal.pone.0286207 (PMC10284388; doi:10.1371/journal.pone.0286207)
Supplement: S1 File — (DOCX) [file pone.0286207.s001.docx]

**S1 File:**

Occupational groupings

**Clinical occupational groups**

Dietitian

Doctor

Healthcare Assistant/Nursing Assistant

Healthcare Scientist (laboratory, technician)

Medical Associate/Assistant Professions

Midwife

Nurse

Occupational Therapist

Paramedic/Ambulance Service

Pharmacist/Pharmacy Technician

Physiotherapist

Psychologist/Assistant Psychologist

Radiographer

Speech & Language Therapist

Ward Manager

Other

**Non-clinical occupational groups**

Administrative and Clerical

Catering Services

Chaplaincy

Clinical Support

Domestic Services

Estates Services

Finance

Healthcare Scientists (laboratory, technician)

Human Resources

IT Support

Management

Research / Academic

Social Services (social worker)

Support Services (driver, porter, security, stores)

Other
