# Supplementary material for: Suicidal thoughts and behaviour among healthcare workers in England during the COVID-19 pandemic: A longitudinal study
Source: PLoS One. 2023 Jun 21;18(6):e0286207. doi: 10.1371/journal.pone.0286207 (PMC10284388; doi:10.1371/journal.pone.0286207)
Supplement: S3 File — (DOCX) [file pone.0286207.s003.docx]

**S3 File:**

Sensitivity analysis adjusting for PHQ9 in models (in Table 5) examining the association between baseline occupational factors and suicidal-related outcomes at Time 1 and Time 2

|  | | **Time 1** | | | | | | **Time 2** | | | | | |
| --- | --- | --- | --- | --- | --- | --- | --- | --- | --- | --- | --- | --- | --- |
| **Occupational factors at Time 1** | **Category** | **Suicidal ideation**  **(aOR; 95% CI)** | | **Suicide attempts**  **(aOR; 95% CI)** | | **Non-suicidal self-injury**  **(aOR; 95% CI)** | | **Suicidal ideation**  **(aOR; 95% CI)** | | **Suicide attempts**  **(aOR; 95% CI)** | | **Non-suicidal self-injury**  **(aOR; 95% CI)** | |
|  |  | **Clinical** | **Non-clinical** | **Clinical** | **Non-clinical** | **Clinical** | **Non-clinical** | **Clinical** | **Non-clinical** | **Clinical** | **Non-clinical** | **Clinical** | **Non-clinical** |
| **Redeployment** | No (Ref) | 1.00 | 1.00 | 1.00 | 1.00 | 1.00 | 1.00 | 1.00 | 1.00 | 1.00 | 1.00 | 1.00 | 1.00 |
|  | Yes | 1.10 (0.77, 1.58) | 1.01 (0.62, 1.65) | 1.48 (0.77, 2.84) | 1.10 (0.32, 3.80) | 1.34  (0.96, 1.89) | 1.48 (0.61, 3.61) | 1.08 (0.70, 1.68) | 1.48 (0.99, 2.22) | 0.73 (0.27, 1.97) | 1.85 (0.81, 4.24) | 0.83 (0.51, 1.36) | 1.35 (0.67, 2.70) |
| **Raising safety concerns** | Confident (Ref) | 1.00 | 1.00 | 1.00 | 1.00 | 1.00 | 1.00 | 1.00 | 1.00 | 1.00 | 1.00 | 1.00 | 1.00 |
|  | Lack of confidence | **1.89 (1.38, 2.58)** | 1.61 (0.90, 2.88) | 0.93 (0.44, 1.95) | 1.32 (0.63, 2.76) | 0.93  (0.53, 1.65) | **2.20 (1.11, 4.33)** | 1.37 (0.86, 2.17) | 1.67 (0.64, 4.36) | 0.55 (0.21, 1.47) | 0.55 (0.08, 3.69) | 1.02 (0.43, 2.44) | 0.98 (0.36, 2.69) |
| **Safety concerns being addressed** | Confidence (Ref) | 1.00 | 1.00 | 1.00 | 1.00 | 1.00 | 1.00 | 1.00 | 1.00 | 1.00 | 1.00 | 1.00 | 1.00 |
|  | Lack of confidence | **1.74 (1.28, 2.37)** | 1.46 (1.00, 2.13) | 1.04 (0.64, 1.69) | 0.67 (0.31, 1.44) | 1.24  (0.84, 1.82) | 1.05 (0.36, 3.05) | **1.33 (1.02, 1.73)** | 1.23 (0.59, 2.56) | 0.98 (0.53, 1.80) | 2.30 (0.80, 6.60) | 1.38 (0.78, 2.41) | 2.43 (0.89, 6.61) |
| **PPE access** | Access (Ref) | 1.00 | 1.00 | 1.00 | 1.00 | 1.00 | 1.00 | 1.00 | 1.00 | 1.00 | 1.00 | 1.00 | 1.00 |
|  | Lack of access | 1.11 (0.69, 1.77) | 0.97 (0.70, 1.33) | 0.43 (0.10, 1.83) | 1.44 (0.69, 2.99) | 0.83  (0.27, 2.53) | 1.47 (0.62, 3.50) | 1.37 (0.91, 2.07) | 1.48 (0.76, 2.87) | 0.50 (0.10, 2.35) | 2.96 (0.97, 9.07) | 0.63 (0.21, 1.90) | 2.37 (0.88, 6.39) |
| **Managerial support** | Supported (Ref) | 1.00 | 1.00 | 1.00 | 1.00 | 1.00 | 1.00 | 1.00 | 1.00 | 1.00 | 1.00 | 1.00 | 1.00 |
|  | Unsupported | **1.58 (1.19, 2.10)** | 1.16 (0.75, 1.80) | 1.12 (0.70, 1.80) | 0.74 (0.39, 1.42) | 1.22  (0.78, 1.90) | 1.00 (0.41, 2.43) | 0.98 (0.67, 1.45) | 0**.**89 (0.60, 1.33) | 0.52 (0.20, 1.38) | 1.27 (0.56, 2.86) | 0.68 (0.27, 1.71) | 1.26 (0.62, 2.60) |
| **Standard of care provided** | Not reduced (Ref) | 1.00 | N/a | 1.00 | N/a | 1.00 | N/a | 1.00 | N/a | 1.00 | N/a | 1.00 | N/a |
|  | Reduced | 1.14 (0.91, 1.41) | N/a | 1.06 (0.67, 1.67) | N/a | 0.92  (0.58, 1.46) | N/a | 1.03 (0.76, 1.39) | N/a | 0.69 (0.29, 1.63) | N/a | 0.82 (0.53, 1.26) | N/a |
| **Potentially morally injurious events** | No exposure (Ref) | 1.00 | 1.00 | 1.00 | 1.00 | 1.00 | 1.00 | 1.00 | 1.00 | 1.00 | 1.00 | 1.00 | 1.00 |
|  | Exposure | 1.22 (1.00-1.50) | 1.00 (0.67, 1.50) | 0.96 (0.64, 1.44) | 0.62 (0.37, 1.02) | 1.18  (0.83, 1.69) | 1.03 (0.58, 1.81) | 1.30 (0.74, 2.30) | 1.60 (0.90, 2.84) | 0.85 (0.31, 2.32) | 1.40 (0.65, 3.03) | 1.10 (0.61, 1.98) | 1.56 (0.83, 2.94) |

Statistically significant results are in bold

aOR: adjusted odds ratios – adjusted for age, sex, ethnicity, PHQ-9, and date of survey completion (6-month data also adjusted for the corresponding outcome measured at Time 1); CI: confidence intervals
