# Supplementary material for: Suicidal thoughts and behaviour among healthcare workers in England during the COVID-19 pandemic: A longitudinal study
Source: PLoS One. 2023 Jun 21;18(6):e0286207. doi: 10.1371/journal.pone.0286207 (PMC10284388; doi:10.1371/journal.pone.0286207)
Supplement: S4 File — (DOCX) [file pone.0286207.s004.docx]

**S4 File:**

Cross-sectional analyses at Time 2 of association between HCW demographic characteristics and suicidal ideation, suicide attempts, and non-suicidal self-injury, stratified by occupational role (i.e., without adjustment for corresponding outcome at Time 1)

| **Demographic characteristics** | **Category** | **Suicidal ideation**  **(aOR; 95% CI)** | | **Suicide attempts**  **(aOR; 95% CI)** | | **Non-suicidal self-injury**  **(aOR; 95% CI)** | |
| --- | --- | --- | --- | --- | --- | --- | --- |
|  |  | **Clinical** | **Non-clinical** | **Clinical** | **Non-clinical** | **Clinical** | **Non-clinical** |
| **Age in years** | **≤30 (Ref)** | 1.00 | 1.00 | 1.00 | 1.00 | 1.00 | 1.00 |
|  | **31-40** | **0.55 (0.40, 0.75)** | **0.41 (0.23, 0.74)** | 0.85 (0.35, 2.08) | 0.53 (0.19, 1.48) | 1.07 (0.58, 1.99) | **0.32 (0.12, 0.84)** |
|  | **41-50** | **0.59 (0.42, 0.83)** | **0.43 (0.27, 0.70)** | 1.30 (0.57, 2.99) | 0.54 (0.24, 1.21) | 0.97 (0.46, 2.08) | **0.49 (0.28, 0.87)** |
|  | **51-60** | **0.38 (0.26, 0.55)** | **0.27 (0.17, 0.42)** | 1.05 (0.45, 2.44) | 0.49 (0.23, 1.05) | 0.62 (0.28, 1.36) | **0.34 (0.18, 0.64)** |
|  | **≥61** | 0.65 (0.41, 1.02) | **(0.30, 0.17, 0.51)** | 1.95 (0.70, 5.45) | 0.91 (0.29, 2.87) | 1.20 (0.43, 3.36) | 0.60 (0.28, 1.26) |
| **Sex** | **Female (Ref)** | 1.00 | 1.00 | 1.00 | 1.00 | 1.00 | 1.00 |
|  | **Male** | 1.16 (0.66, 2.01) | 1.31 (0.86, 1.98) | 1.80 (1.08, 3.00) | 0.62 (0.16, 2.32) | 1.10 (0.63, 1.91) | 0.63 (0.27, 1.49) |
| **Ethnicity** | **White (Ref)** | 1.00 | 1.00 | 1.00 | 1.00 | 1.00 | 1.00 |
|  | **Black/African/Caribbean/Black British** | 0.43 (0.15, 1.17) | 0.71 (0.23, 2.13) | 0.98 (0.30, 3.24) | 0.65 (0.07, 6.05) | 0.84 (0.34, 2.08) | 0.46 (0.05, 4.05) |
|  | **Asian/Asian British** | 1.03 (0.63, 1.71) | **0.48 (0.30, 0.79)** | 1.70 (0.62, 4.70) | 1.41 (0.66, 3.02) | 1.03 (0.40, 2.60) | 1.05 (0.42, 2.62) |
|  | **Mixed/Multiple racial and ethnic groups** | 1.11 (0.34, 3.66) | 0.96 (0.18, 5.04) | 2.45 (0.78, 7.68) | 0.09 (0.01, 1.01) | 2.38 (1.11, 5.08) | 1.12 (0.21, 5.90) |
|  | **Other racial and ethnic minority groups** | - | 0.25 (0.02, 3.04) | - | - | - | - |

Statistically significant results are in bold

aOR: adjusted odds ratios – adjusted for age, sex, ethnicity, and date of survey completion; CI: confidence intervals
