# Supplementary material for: Suicidal thoughts and behaviour among healthcare workers in England during the COVID-19 pandemic: A longitudinal study
Source: PLoS One. 2023 Jun 21;18(6):e0286207. doi: 10.1371/journal.pone.0286207 (PMC10284388; doi:10.1371/journal.pone.0286207)
Supplement: S5 File — (DOCX) [file pone.0286207.s005.docx]

**S5 File:**

Cross-sectional analyses at Time 2 of association between HCW occupational factors and suicidal ideation, suicide attempts, and non-suicidal self-injury, stratified by occupational role (i.e., including occupational factors measured at Time 2 and not adjusting for the corresponding outcome at Time 1)

| **Occupational factor at Time 2** | **Category** | **Suicidal ideation**  **(aOR; 95% CI)** | | **Suicide attempts**  **(aOR; 95% CI)** | | **Non-suicidal self-injury**  **(aOR; 95% CI)** | |
| --- | --- | --- | --- | --- | --- | --- | --- |
|  |  | **Clinical** | **Non-clinical** | **Clinical** | **Non-clinical** | **Clinical** | **Non-clinical** |
| **PPE access** | Access (Ref) | 1.00 | 1.00 | 1.00 | 1.00 | 1.00 | 1.00 |
|  | Lack of access | 1.33 (0.64, 2.73) | **3.97 (1.63, 9.67)** | 0.43 (0.07, 2.50) | **10.27 (3.57, 29.58)** | 1.43 (0.42, 4.82) | **7.95 (3.46, 18.29)** |
| **Managerial support** | Supported (Ref) | 1.00 | 1.00 | 1.00 | 1.00 | 1.00 | 1.00 |
|  | Unsupported | **1.84 (1.33, 2.54)** | **2.17 (1.49, 3.15)** | 0.62 (0.26, 1.50) | 1.14 (0.66, 1.97) | 1.22 (0.70, 2.13) | 1.61 (0.87, 2.97) |
| **Standard of care provided** | Not reduced (Ref) | 1.00 | N/a | 1.00 | N/a | 1.00 | N/a |
|  | Reduced | 1.72 (0.91, 3.26) | N/a | 1.50 (0.54, 4.15) | N/a | 1.95 (0.81, 4.71) | N/a |
| **Potentially morally injurious events** | No exposure (Ref) | 1.00 | 1.00 | 1.00 | 1.00 | 1.00 | 1.00 |
|  | Exposure | **2.47 (1.81, 3.38)** | **2.64 (1.71, 4.08)** | 0.80 (0.50, 1.29) | 1.17 (0.63, 2.17) | 1.21 (1.00, 1.47) | 1.76 (0.91, 3.42) |

Statistically significant results are in bold

aOR: adjusted odds ratios – adjusted for age, sex, ethnicity, and date of survey completion; CI: confidence intervals

Questions about safety concerns and re-deployment were not included in the survey at Time 2
