# Supplementary material for: Suicidal thoughts and behaviour among healthcare workers in England during the COVID-19 pandemic: A longitudinal study
Source: PLoS One. 2023 Jun 21;18(6):e0286207. doi: 10.1371/journal.pone.0286207 (PMC10284388; doi:10.1371/journal.pone.0286207)
Supplement: S6 File — (DOCX) [file pone.0286207.s006.docx]

**S6 File:**

Number and proportion of HCWs reporting exposure to occupational factors and probable depression at Time 1, stratified by occupational group (includes missing data)

| Variable | Category |  | No. (%) unless otherwise specified | |
| --- | --- | --- | --- | --- |
|  |  | **Overall**  **(n=12,514)** | **Clinical**  **(n=7,847)** | **Non-clinical**  **(n=4,571)** |
| Redeployment | *No (Ref)* | 11,009 (87.9) | 6,822 (86.9) | 4,102 (90.2) |
|  | *Yes* | 1,472 (11.8) | 1,009 (12.9) | 455 (9.5) |
|  | *Missing* | 33 (0.3) | 16 (0.2) | 14 (0.3) |
| PPE access | *Access (Ref)* | 9,752 (78.8) | 6,628 (85.6) | 3,051 (62.9) |
|  | *Lack of access* | 967 (9.1) | 585 (8.6) | 376 (10.2) |
|  | *Missing* | 1,795 (12.2) | 634 (5.8) | 1,144 (26.9) |
| Managerial support | *Supported (Ref)* | 10,272 (80.2) | 6,397 (79.3) | 3794 (82.4) |
|  | *Unsupported* | 2,192 (19.4) | 1,428 (20.4) | 752 (17.1) |
|  | *Missing* | 50 (0.4) | 22 (0.3) | 25 (0.6) |
| Raising safety concerns | *Confident (Ref)* | 10,536 (83.6) | 6,718 (85.1) | 3,745 (80.3) |
|  | *Lack of confidence* | 958 (8.0) | 691 (8.9) | 260 (6.0) |
|  | *Missing* | 1,020 (8.4) | 438 (6.1) | 566 (13.7) |
| Safety concerns being addressed | *Confident (Ref)* | 9,995 (78.5) | 6,252 (78.5) | 3,675 (78.5) |
|  | *Lack of confidence* | 1,491 (13.1) | 1,148 (15.4) | 331 (7.8) |
|  | *Missing* | 1,028 (8.4) | 447 (6.1) | 565 (13.7) |
| Standard of care provided | *Not reduced (Ref)* | 8,623 (71.2) | 6,150 (80.0) | 2,414 (51.9) |
|  | *Reduced* | 1,640 (12.9) | 1,352 (15.7) | 281 (6.4) |
|  | *Missing* | 2,251 (16.0) | 345 (4.8) | 1,876 (41.7) |
| Potentially morally injurious events | *No exposure (Ref)* | 8,804 (67.6) | 5,369 (66.2) | 3,366 (70.9) |
|  | *Exposure* | 2,898 (25.4) | 1,951 (26.6) | 931 (22.7) |
|  | *Missing* | 812 (7.0) | 527 (7.2) | 274 (6.4) |
| Probable depression  (PHQ-9 score ≥10) | *No (Ref)* | 8,650 (68.1) | 5,458 (68.5) | 3,127 (67.1) |
|  | *Yes* | 3,259 (26.8) | 1,999 (26.3) | 1,237 (27.9) |
|  | *Missing* | 605 (5.2) | 390 (5.2) | 206 (5.0) |

n=frequencies (unweighted raw data), percentages are weighted
