# Supplementary material for: Suicidal thoughts and behaviour among healthcare workers in England during the COVID-19 pandemic: A longitudinal study
Source: PLoS One. 2023 Jun 21;18(6):e0286207. doi: 10.1371/journal.pone.0286207 (PMC10284388; doi:10.1371/journal.pone.0286207)
Supplement: Attachment — Submitted filename: Peer review_PLOS ONE.docx [file pone.0286207.s007.docx]

I would like to thank you for giving me the chance to review this manuscript. The paper reports a large prospective study on incidence risk and prevalence of suicidal thoughts and behavior, and their relationship with demographic and occupational risk factors. The paper is well organized and clearly written. Nevertheless, there are some methodological issues that need to be addressed. My comments on the different sections of the paper are presented below.

**Abstract**

- May be useful to include the type of design used in the title
- The authors argued that there has been speculation regarding increase risk in suicide among healthcare workers during COVID-19. Are there no any preliminary empirical evidence on this?
- Is there any particular reason to stratify results by occupational role? I thought that this can be included in the regression model as a variable.
- It is not clear whether the design is longitudinal or repeated cross-sectional in the abstract
- I would suggest to highlight the baseline findings in the abstract
- I would suggest conclusion instead of discussion in the abstract.
- There is inconsistency between the results and conclusion sub-section s. While the results section highlights prevalence and incidence, the conclusion emphasized on factors associated with the outcome variables.

**Background**

- The authors claimed “Many studies from across the world have investigated the impact of the pandemic on suicidal thoughts and behavior.” However, they did not summarize what these studies found out.
- There is need to synthesize the findings of previous studies in the area (i. e. the prevalence of suicide thoughts and behaviors and contributing factors during the COVID-19 pandemic and other related pandemics). The authors need to justify this is worth studying.
- Overall, the background section is a bit shallow and doesn’t comprehensively synthesize the existing evidence and indicate the research gap.

**Methods**

- The authors started the methods section with describing the online survey. I would suggest this to come after describing about the design of the study, the study context and participants.
- Would 5% response rate be acceptable? I would say that those who have STB are likely to complete the survey more than those who have not and this is likely to overestimate the prevalence and incidence reported in this study.
- The psychometric properties and acceptability and feasibility of the three questions used to measures STB are not described. Did previous studies use these questions? Are they validated? What are the item response characteristics or qualities of these questions?
- The authors indicated that they used two-level random-effects logistic regression models. Is this a type of multilevel analysis? From the description of the analysis procedures, one can understand the need for a multilevel analysis (Trust is included in the model as explanatory variable and HCWs are clustered within each Trust).

**Results**

- There seems inconsistency in reporting the response rate: 5% in the methods section and 16% in the results section
- It is not clear what the authors are meant by the Time1 full cohort. It possible to understand from the data presented that only Time 1 sample and Time 2 sample responded to questions related to STB. I don’t think the Time 1 full cohort is related to any of the analyses done in this study.
- Is there any rationale for the age categories used in this study?
- I would suggest organizing the results section into subjects (such as prevalence, incidence risk, factors associated etc….). This will make easy for the reader to follow.
- It is not clear why separate models were estimated for the socio-demographic characteristics and occupational factors. For the same outcomes, estimating several models will increase the type error.
- It is necessary to make explicit (in the methods section) the fact that cross-sectional analyses were done for investigating the relationship between socio-demographic/occupational characteristics and STB.

**Discussion**

- The authors claimed that they did prospective relationship between socio-demographic and occupational factors and STB (Page 17, line 303). I don’t think this is correct. I didn’t see any prospective association analyses. Time 1 and Time 2 reports of STB are separately associated with socio-demographic and occupational factors.
- The findings of the study reported in this paper and compared with several previous studies conducted in different parts of the world. However, these studies are not synthesized and reported in the background section to give context to the problem and indicate the research gap.
